# Signature literature review reveals *AHCY*, *DPYSL3*, and *NME1* as the most recurrent prognostic genes for neuroblastoma

**DOI:** 10.1186/s13040-023-00325-1

**Published:** 2023-03-04

**Authors:** Davide Chicco, Tiziana Sanavia, Giuseppe Jurman

**Affiliations:** 1 Institute of Health Policy Management and Evaluation, University of Toronto, 155 College Street, M5T 3M7 Toronto, Ontario, Canada; 2 Dipartimento di Scienze Mediche, Università di Torino, Via Verdi 8, 10124 Turin, Italy; 3 Data Science for Health Unit, Fondazione Bruno Kessler, Via Sommarive 18, 38123 Povo (Trento), Italy

**Keywords:** Signatures, Prognostic signatures, Neuroblastoma, Neuro-oncology, Scientific literature, Review, Survey, Pediatric cancers, Endocrine neoplasia

## Abstract

Neuroblastoma is a childhood neurological tumor which affects hundreds of thousands of children worldwide, and information about its prognosis can be pivotal for patients, their families, and clinicians. One of the main goals in the related bioinformatics analyses is to provide stable genetic signatures able to include genes whose expression levels can be effective to predict the prognosis of the patients. In this study, we collected the prognostic signatures for neuroblastoma published in the biomedical literature, and noticed that the most frequent genes present among them were three: *AHCY*, *DPYLS3*, and *NME1*. We therefore investigated the prognostic power of these three genes by performing a survival analysis and a binary classification on multiple gene expression datasets of different groups of patients diagnosed with neuroblastoma. Finally, we discussed the main studies in the literature associating these three genes with neuroblastoma. Our results, in each of these three steps of validation, confirm the prognostic capability of *AHCY*, *DPYLS3*, and *NME1*, and highlight their key role in neuroblastoma prognosis. Our results can have an impact on neuroblastoma genetics research: biologists and medical researchers can pay more attention to the regulation and expression of these three genes in patients having neuroblastoma, and therefore can develop better cures and treatments which can save patients’ lives.

**Supplementary Information:**

The online version contains supplementary material available at 10.1186/s13040-023-00325-1.

## Introduction

Neuroblastoma is a type of brain tumor that affects thousands of newborns and young children worldwide [1]. Neuroblastoma almost always develops before age 5 and rarely develops in children over age 10, and it can be of low-, middle- or high-risk. The therapy for patients with high-risk neuroblastoma includes combinations of chemotherapy, surgery, high-dose chemotherapy with stem cell rescue (also known as autologous stem cell transplantation), radiation and immunotherapy [1].

Most of the times, neuroblastoma happens as a result of gene expression changes in neuroblasts that happen during the child’s development, sometimes even before birth, and the causes of these changes are still unknown [2]. To this end, discovering which genes have a prognostic role in neuroblastoma is fundamental to understand the pathogenesis and the disease progression, but also for treatment decisions on potential targeted therapies.

Genetic signatures are lists of genes that have an important prognostic or diagnostic role in patients with a specific disease: a strong prognostic signature for neuroblastoma, for example, contains a list of genes whose change of expression levels can be used in a model able to predict the survival risk of new patients.

Genetic signatures can be derived from microarray gene expression [3], RNA-Seq gene expression [4], but also from other regulatory gene expression elements like microRNA, DNA methylation [5] and other biomolecular data.

Despite their usefulness, genetic signatures of the same diseases can have low overlap between each other: signatures in fact might contain false positive genes, that are genes having a strong signal in an obsolete or noisy technology and therefore included in the signature, but actually are unrelated to that particular disease [6]. Integrating and comparing signatures of the same disease published in the literature can be a way to address this problem.

In this study, we queried the scientific literature and found ten neuroblastoma prognostic signatures [7–16], we decided to extrapolate the most common genes and investigate their role in neuroblastoma. Only three genes resulted as the most common and shared in a limited number of signatures: AHCY, DPYSL3, and NME1. This result confirms the lack of reproducibility among the prognostic genetic signatures for neuroblastoma. We then performed a survival analysis considering the largest available neuroblastoma gene expression datasets in Gene Expression Omnibus (GEO) [17] and ArrayExpress [18] in order to understand the behavior of these three genes in relation to *time-to-event* data. Moreover, we tested the predictive power of these three genes by employing a machine learning algorithm on a binary dataset containing data of survived and deceased patients. Finally, we performed a thorough literature validation considering all the articles that show a correlation between these three genes and neuroblastoma treatments.

To the best of our knowledge, no previous studies have used the scientific literature to detect the most recurrent prognostic genes for neuroblastoma.

We organize the rest of this article as follows. After this Introduction, we explained the methods for genes and datasets retrieval, the methods for survival analysis, the methods for the machine learning analysis, and the methods for literature validation in section Methods, and we then report the results of these phases in section Results. Eventually, we discuss the impact and the consequences of our results and report some limitations (section Discussion).

## Methods

### Prognostic genes discovery and dataset search

We retrieved the most recurrent neuroblastoma prognostic genes by comparing the prognostic signatures currently available in the scientific literature (Subsection S1.1). We included the neuroblastoma signatures indicated for survival and prognosis, and we excluded the diagnostic ones. We then converted each gene symbol into Ensembl ID’s through g:Convert of g:Profiler [19]. We immediately noticed both the limited number of studies providing a curated signature list of prognostic genes and the low overlap between the these lists, that motivated us for the selection of the the most recurrent genes.

For the machine learning validation phase on gene expression, we searched for neuroblastoma gene expression datasets on Gene Expression Omnibus (GEO) [17] through geoCancerPrognosticDatasetsRetriever [20] having the survived-deceased binary label for each patient.

We found and downloaded one gene expression dataset through GEOquery [21] and BioMart [22]: the Hiyama2010 dataset coded as GSE16237 [23] on GEO. We performed the gene probeset-symbol annotation done through geneExpressionFromGEO [24] and Jetset [25]. On the dataset found, we performed the batch correction [26] through limma [27]. We used this Hiyama2010 dataset for the machine learning validation phase and not for the survival analysis phase (next subsection) because it does not contain the temporal component and because we prefered to keep these two validation phases independent from each other.

### Validation of risk and hazard ratio in multi-studies overall survival data

To validate the robustness of the most recurrent genes resulting from the literature search (Subsection Prognostic genes discovery and dataset search) as potential prognostic biomarkers, we first investigated the pooled risk ratio (RR) based on the number of deaths in neuroblastoma associated with high/low expression of each gene. Specifically, for this survival analysis phase we manually searched for gene expression datasets on Gene Expression Omnibus [17], ArrayExpress [18] and R2 database [28] with the survived status and the survival time feature for each feature.

Only large datasets, with at least 80 samples, providing pre-processed and normalized gene expression values with associated overall survival data were included. A $RR>1$ represents poorer prognosis for the higher expression group, whereas a $RR<1$ indicates poorer prognosis for the lower expression group. Each gene was first analyzed separately, and its expression was classified in a binary way, considering the expression values below the 25$^{th}$ or above the 75$^{th}$ percentile to identify the samples at low or high expression of that gene, respectively.

Then, a combination of the genes extracted by the literature search was also evaluated: a consensus score was generated considering the average of the ranking positions obtained for each gene expression (increasingly ordered), assigning the same ranking position to ties and inverting the ranking of the genes showing $RRs<1$. In this case, the scores belonging to the first and last quartiles were classified as “low” and “high”, respectively. Both fixed- and random-effects models using Mantel-Haenszel [29] and restricted maximum-likelihood [30] estimators were considered, respectively. Heterogeneity across studies was assessed by the Cochrane $I^2$ metric [31] and chi-squares statistics. Chi-squared *p*-values $<0.1$ and an $I^2$ values $>50$% were associated to statistically significant heterogeneity. Potential publication bias was evaluated using Begg and Mazumdar’s test [32] and Begg’s funnel plot [33], considering *p*-values $<0.05$ to indicate statistical bias.

Afterwards, we considered the number of deaths in relation with the follow-up time and evaluated the expression values of the genes and their consensus score through Cox proportional hazard model [34], adjusted by the age of the patients. This confounding factor was discretized into two classes, using a threshold of 18 months. The hazard ratios (HRs) with 95% confidential intervals (CIs) for overall survival observed along a follow-up time were estimated by the model. An HR $>1$ implied poorer survival for the higher expression group. In contrast, an HR$<1$ implied poorer survival for the lower expression group. Both Wald test applied to the coefficient of the model used to estimate the HR and Log-rank test were considered for the evaluation. *p*-values less than 0.05 were considered statistically significant. All statistical analyses were performed using R programming language version 4.1.2. The R packages meta (version 5.2), dmetar (version 0.9) and survival (version 3.2-13) were used for the meta-analysis of the RRs and the survival analysis through Cox proportional hazard model, respectively.

### Binary classification method

To further verify the predictive power of our three proposed genes, we decided to use them for a prognostic binary classification based on Random Forests [35] machine learning method, on an alternative dataset not employed for the previous phases of the analysis. We downloaded the Hiyama2010 dataset from GEO (GSE16237 [23]), which contains microarray gene expression of patients diagnosed with neuroblastoma. In this cohort there are 12 deceased patients and 39 survived patients, that means 23.53% negative data instances and 76.47% data instances. We decided to employ this external, alternative dataset we did not use for the other validation steps, to make our analysis as robust as possible [36].

After downloading this dataset, we applied a batch correction method through the limma [27] package of Bioconductor [37] to remove the noise of the batch effects from the microarray data [26]. We then selected only the three gene profiles of the probesets of our three proposed prognostic genes in the HG-U133_Plus_2] Affymetrix Human Genome U133 Plus 2.0 Array (GPL570) microarray platform: 200903_s_at (AHCY), 201431_s_at (DPYSL3), and 201577_at (NME1). Afterwards, we applied Random Forests [35] by splitting the dataset into training (80% randomly selected patients’ profiles) and test (20% remaining patients’ profiles) sets [38]. We repeated the execution 100 times and generated confusion matrices on the tests sets, that we assessed through traditional rates such as the Matthews correlation coefficient (MCC) [39], ROC AUC and others (Table 5), recorded as average values and standard deviations. We highlighted the result measured through the MCC [40–42].

### Validation on the literature

We investigated the role of AHCY, DPYSL3, and NME1 genes in the scientific literature by looking for studies involving at least one of these three genes and neuroblastoma in Google Scholar [43] on 20th February 2022.

We retrieved the aliases of the names of these three genes on GeneCards.org [44–46]:

- AHCY aliases: SAHH, S-Adenosyl-L-Homocysteine Hydrolase, S-Adenosylhomocysteine Hydrolase.
- DPYSL3 aliases: CRMP4, CRMP-4, ULIP, DRP-3, DRP3, ULIP1.
- NME1 aliases: NM23-H1, NM23-H1, NDPKA, NM23.

For each of this term, we made a search on Google Scholar [43] including the neuroblastoma keyword (for example, “AHCY neuroblastoma”, “SAHH neuroblastoma”, etc.) and recorded all the scientific studies describing an active role of the gene and neuroblastoma survival or prognosis.

## Results

### Prognostic genes found

In our literature search, we found ten neuroblastoma prognostic signatures, whose quantitative characteristics and references are reported in Table 1 and in Supplementary Subsection S1.2.

Three genes resulted being more present than the others in three signatures (Table 2): AHCY, DPYSL3, and NME1.

The probability that a three-genes triple occur in three different signatures can be experimentally estimated as $P\approx 5\cdot 10^{-8}$. In fact, randomly rearranging the 300 genes included in the 10 signatures in the same setup of the current situation, and repeating such experiments for *N* runs, three genes occur in three different signatures in $\alpha \cdot N$ runs, with $\alpha \approx 0.24$ in average. Since there are $$T=\left( {\begin{array}{c}300\\ 3\end{array}}\right) =4455100$$ distinct sets of three genes, the probability *P* can be evaluated as $P=\frac{\alpha }{T}\approx 5\cdot 10^{-8}$. This yields that random effects are quite unlikely responsible for the selection of the investigated triple AHCY, DPYSL3, and NME1.

This aspect confirms the low overlap between gene lists of the neuroblastoma prognostic signatures found: among 10 signatures, only 3 gene lists share 3 common genes, which are the most frequent elements among the lists (Subsection S1.2). Surprisingly, the famous neuroblastoma-related MYCN gene is not among the most frequent genes [47]. It is present only in two signatures [10, 11] out of ten.

**Table 1:** List of neuroblastoma prognostic signatures found in the literature. Article: reference. # genes: number of genes. We reported the lists of the genes of these signatures in Supplementary Subsection S1.2

	Publication	
article	year	# genes
Vermeulen et al. [10]	2009	59
De Preter et al. [11]	2010	42
Garcia et al. [14]	2012	3
Valentijn et al. [12]	2012	157
Frumm et al. [15]	2013	59
Zhong et al. [13]	2018	4
Wang et al. [16]	2020	5
Cangelosi et al. [7]	2020	6
Jin et al. [9]	2020	9
Zhong et al. [8]	2021	5

**Table 2:** Most recurrent prognostic genes for neuroblastoma. For each gene, we report its symbol, its Ensembl ID, and its complete gene name on GeneCards.org. The signatures column indicates the references of the neuroblastoma signatures which contain each gene

Gene symbol	Ensembl gene ID	Gene name	Signatures
AHCY	ENSG00000101444	Adenosylhomocysteinase	[8, 10, 11]
DPYSL3	ENSG00000113657	Dihydropyrimidinase Like 3	[10, 11, 15]
NME1	ENSG00000239672	NME/NM23 Nucleoside Diphosphate Kinase 1	[10, 11, 14]

### Computational evaluation of the impact of the most recurrent prognostic genes across the largest neuroblastoma studies

Both array-based and sequencing-based data were considered. A description of the selected datasets, together with the information related to the features considered for the three genes of interest (AHCY, DPYSL3, NME1) is reported in Table 3. Some of these datasets were employed (as training set or test set) in the studies identifying the ten neuroblastoma signatures we found in the literature (Supplementary Table 1), but these studies leverage other datasets, too, to verify the effectiveness of their proposed signatures. The overlap between the set of datasets used by these ten signature studies and the set of dataset we used for our survival analysis and binary classification phases is always lower than 50% (Supplementary Table 1).

**Fig. 1:**
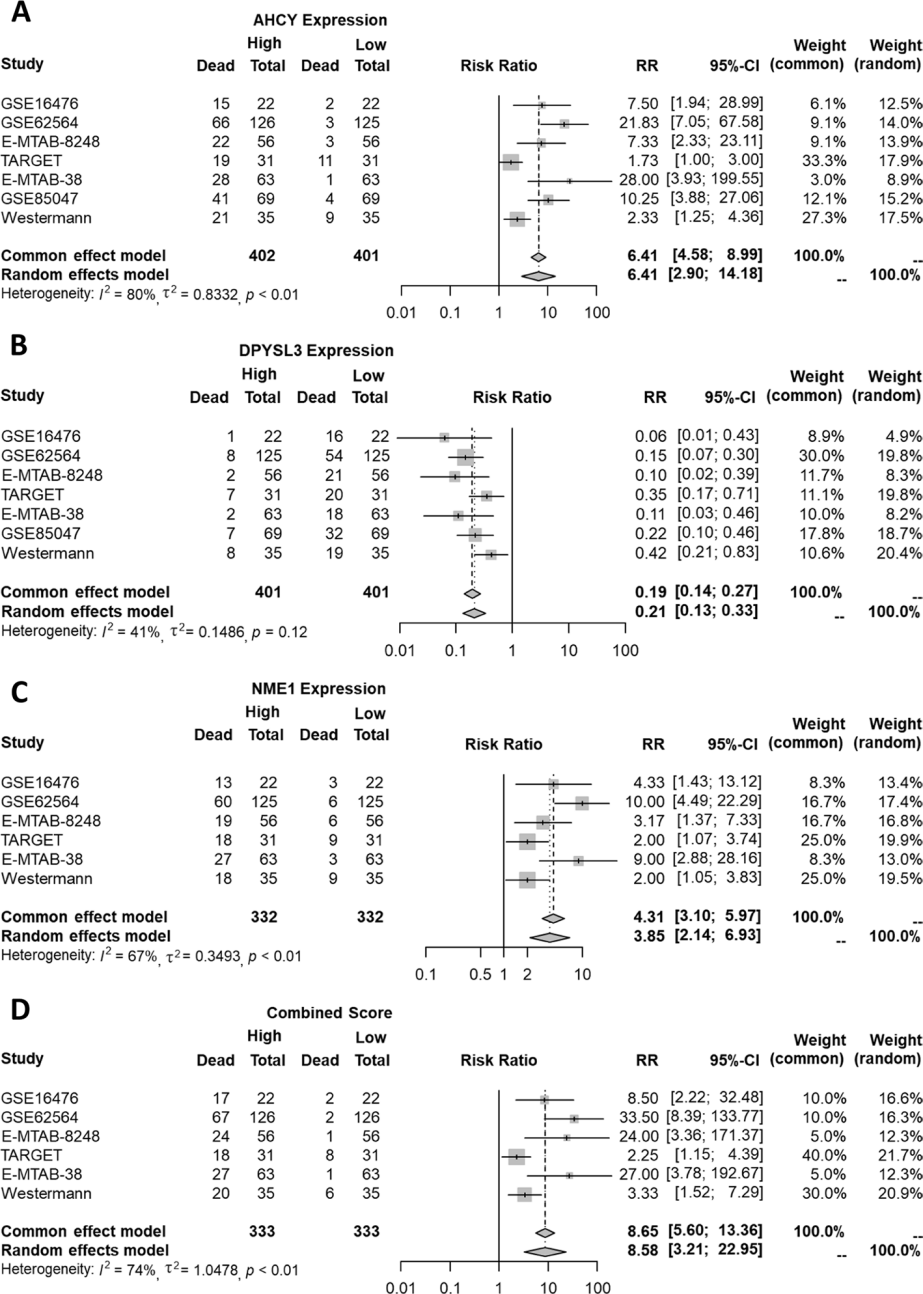
Forest plot of the association between associating low/high gene expression with and all-cause mortality. Forest plots displaying the results from the meta-analysis for the three genes considered separately, i.e. AHCY (A), DPYSL3 (B), NME1 (C), and for the score which combines the expression-based ranks of the three genes, considering the reversed rank for DPYSL3 since it showed RRs $<1$. Results from both fixed- (common) and random-effects models are reported. Abbreviations: RR, Risk Ratio; CI, Confidence Interval

The expression of all the three genes resulted significantly associated with the death rate, as displayed in Fig. 1. Specifically, at higher expression of both AHCY and NME1 higher death rates were observed (considering median percentages across all the studies, 55.9% at high versus 5.8% at low AHCY expression with estimated RR 6.4 and $p<0.0001$ from Mantel-Haenszel test, while 49.7% at high versus 12.2% at low NME1 expression with estimated RR equal to 3.85 for the random-effects model and to 4.31 for fixed(common)-effect model, obtaining $p<0.0001$ in both models). On the other hand, lower death rates were observed at higher expression of the gene DPYSL3 (considering median percentages across all the studies, 6.4% at high versus 46.4% at low expression with estimated RR 0.2 and $p<0.0001$ from Mantel-Haenszel test).

Combining all the genes, where the ranking of the DPYSL3 expression was inverted to be integrated with the rankings from AHCY and NME1, it is possible to observe an increased pooled RR (8.58 for the random-effects model and 8.65 for fixed(common)-effect model, obtaining $p<0.0001$ in both models with Mantel-Haenszel test), with a median death rate equal to 42.9% at high and to 5.4% at low consensus score. The heterogeneity across the studies ranges between 41% (observed for DPYSL3, $p=0.12$) and 80% (observed for AHCY, $p<0.01$). However, funnel plots inspection (Supplementary Fig. S1) and Begg and Mazumdar’s test results indicate the presence of funnel plot asymmetry, showing that the publication bias was unlikely (all *p*-values $>0.05$).

Afterwards, we investigated the time-dependent risk of death in terms of hazard ratios (adjusted for the age). Results are reported in Table 4. All the Cox models which include both expression and age as confounding factors showed signicant association with overall survival. In 6 over 7 studies for AHCY and DPYSL3, in 4 over 6 studies for NME1 and in 5 over 6 studies for the consensus score the related HR was found significant.

**Table 3:** Datasets used in the meta-analysis. Description of the 7 datasets used in the meta-analysis with the related reference to the study, specifying the size, the array/sequencing platform, the pre-processing approach used to normalize the data and the type of feature (for example, probeset ID, Refseq ID, Ensembl transcript/gene ID) considered to identified each gene analyzed

Dataset	Samples	Platform	Normalization	Features
GSE16476 [48]	88	HG-U133_Plus_2	MAS5.0 algorithm (target signal = 100) using GCOS software (Affymetrix)	AHCY:200903_s_at; DPYSL3:201431_s_at; NME1:201577_at
GSE62564 (SEQC) [49]	498	Illumina HiSeq 2000	Reads per million mapped reads (RPM)	AHCY:NM_000687; DPYSL3:NM_001387; NME1:NM_000269
E-MTAB-8248 [50]	223	Agilent-020382 Human Custom Microarray 44k	preprocessCore (quantile)	AHCY:A_23_P17575; DPYSL3:A_24_P149036; NME1:A_23_P152804
TARGET [51]	161	Illumina Hiseq 2000	Fragments per kilo base of transcript per million mapped fragment (FPKM)	AHCY: ENST00000217426; DPYSL3: ENST00000398514; NME1: ENST00000393196
E-MTAB-38 [52]	251	customized 11K olignucleotide-microarray (Agilent)	Variance Stabilization Normalization (VSN)	AHCY: A-MEXP-255.2721, A-MEXP-255.3013, A-MEXP-255.7928; DPYSL3: A-MEXP-255.617, A-MEXP-255.7551; NME1: A-MEXP-255.8195
GSE85047 [53]	283	Affymetrix Human Exon 1.0 ST Array	Robust Multichip Average (RMA)	AHCY:3903361; DPYSL3:2880292; NME1: not available
Westermann [54]	144	Illumina Hiseq 2000 and Hiseq 4000	Transcripts per million (TPM)	AHCY: ENSG00000101444; DPYSL3: ENSG00000113657; NME1: ENSG00000239672

**Table 4:** Hazard ratios from Cox proportional hazard models testing the prognostic role for all-cause mortality. Results are reported for the three genes considered separately (AHCY, DPYSL3, NME1) and for the score which combines the expression-based ranks of the three genes, considering the reversed rank for DPYSL3 since it showed RRs $<1$. Abbreviations: HR, Hazard Ratio; CI, Confidence Interval; LR, Log-Rank

Gene	Study	HR	HR CI lower	HR CI upper	HR *p*-value	LR test *p*-value
AHCY	GSE16476	1.99	1.29	3.07	0.002	3.56 $\times 10^{-13}$
AHCY	GSE62564	1.59	1.4	1.81	1.43 $\times 10^{-12}$	3.87 $\times 10^{-43}$
AHCY	E-MTAB-8248	1.68	1.35	2.1	3.61 $\times 10^{-06}$	6.25 $\times 10^{-14}$
AHCY	TARGET	1.12	0.883	1.42	0.353	0.004
AHCY	E-MTAB-38	2.49	1.87	3.31	4.96 $\times 10^{-10}$	9.03 $\times 10^{-27}$
AHCY	GSE85047	1.8	1.43	2.26	4.11 $\times 10^{-07}$	1.05 $\times 10^{-19}$
AHCY	Westermann	1.81	1.42	2.31	1.62 $\times 10^{-06}$	7.76 $\times 10^{-08}$
DPYSL3	GSE16476	0.47	0.321	0.689	1.07 $\times 10^{-04}$	2.45 $\times 10^{-14}$
DPYSL3	GSE62564	0.577	0.448	0.745	2.31 $\times 10^{-05}$	3.74 $\times 10^{-28}$
DPYSL3	E-MTAB-8248	0.577	0.38	0.877	0.01	3.35 $\times 10^{-09}$
DPYSL3	TARGET	0.744	0.546	1.01	0.0613	0.001
DPYSL3	E-MTAB-38	0.659	0.486	0.893	0.007	4.87 $\times 10^{-14}$
DPYSL3	GSE85047	0.729	0.592	0.897	0.003	4.30 $\times 10^{-15}$
DPYSL3	Westermann	0.639	0.491	0.832	0.001	4.37 $\times 10^{-05}$
NME1	GSE16476	1.38	0.934	2.04	0.105	1.46 $\times 10^{-11}$
NME1	GSE62564	1.4	1.24	1.57	2.89 $\times 10^{-08}$	4.21 $\times 10^{-35}$
NME1	E-MTAB-8248	1.55	1.17	2.05	0.002	4.22 $\times 10^{-10}$
NME1	TARGET	1.15	0.885	1.48	0.302	0.00319
NME1	E-MTAB-38	2.3	1.72	3.08	2.33 $\times 10^{-08}$	2.34 $\times 10^{-24}$
NME1	Westermann	1.52	1.14	2.03	0.004	1.52 $\times 10^{-04}$
Combined	GSE16476	2.23	1.4	3.55	0.001	9.82 $\times 10^{-14}$
Combined	GSE62564	2.92	2.25	3.79	1.12 $\times 10^{-15}$	1.73 $\times 10^{-41}$
Combined	E-MTAB-8248	2.25	1.55	3.25	1.7E $\times 10^{-05}$	5.79 $\times 10^{-12}$
Combined	TARGET	1.29	0.965	1.72	0.086	0.001
Combined	E-MTAB-38	3.24	2.14	4.92	3.22 $\times 10^{-08}$	8.35 $\times 10^{-22}$
Combined	Westermann	2.04	1.55	2.7	4.71 $\times 10^{-07}$	6.08 $\times 10^{-08}$

Moreover, our Random Forests classifier on the Hiyama2010 obtained ROC AUC = 0.877 and PR AUC = 0.952 in the [0; 1] range, with MCC = +0.517 in the $[-1;+1]$ interval, that means excellent binary classification (Table 5).

**Table 5:** Binary classification results on the Hiyama2010 dataset. We consider only the three genes AHCY, DPYSL3 and NME1. We report the mean scores of 100 executions with corresponding standard deviations, obtained through the Random Forests [35] algorithm. Positives: data of survived patients. Negatives: data of deceased patients. MCC: Matthews correlation coefficient. MCC: worst and minimum value $= -1$ and best and maximum value $= +1$. TP rate: true positive rate, sensitivity, recall. TN rate: true negative rate, specificity. PR: precision-recall curve. PPV: positive predictive value, precision. NPV: negative predictive value. ROC: receiver operating characteristic curve. AUC: area under the curve. F$_1$ score, accuracy, TP rate, TN rate, PPV, NPV, PR AUC, ROC AUC: worst and minimum value $= 0$ and best and maximum value $= 1$. Confusion matrix threshold for TP rate, TN rate, PPV, and NPV: 0.5. Hiyama2010 dataset: GSE16237 [23]

	MCC	F$_1$ score	accuracy	TP rate	TN rate	PPV	NPV	PR AUC	ROC AUC
mean	+0.517	0.885	0.834	0.892	0.647	0.893	0.685	0.952	0.877
std. dev.	0.355	0.096	0.128	0.127	0.329	0.110	0.331	0.080	0.151

### AHCY, DPYSL3, and NME1 in the neuroblastoma literature

In the following text, we report a description of the relevant role of AHCY, DPYSL3, and NME1 in neuroblastoma development highlighted by the studies considered by the state-of-the-art literature, as described in subsection Validation on the literature.

**AHCY and neuroblastoma**

The metabolic enzyme adenosyl-homocysteinase (AHCY) is one of the most conserved enzymes in living organisms. In mammals, AHCY mediates the reversible catalysis of S-adenosylhomocysteine (SAH) to adenosine and L-homocysteine, and it plays a key role in DNA methylation maintenance, thus resulting fundamental during epigenomic reprogramming throughout embryo development and/or in disease progression. In cancer, AHCY seems to have a dual role both as tumor suppressor [55] or tumor promoter, based on the cancer type. Its inhibition for example was linked to anti-migratory and anti-invasive activity of breast cancer cells [51, 56]. In neuroblastoma, literature evidence paint a picture where AHCY is constitutively active in cancer cells, but further enhanced by MYCN. Indeed, AHCY is directly regulated by MYC proteins and like these, associated with poor prognosis of neuroblastoma patients. As a matter of fact, the depletion of MYCN cause the down-regulation of AHCY. Moreover, AHCY inhibition reduces colony formation capacity and glutathione synthesis especially in neuroblastoma cell lines with high MYCN expression. The specific synthetic lethality through genetic or pharmacological AHCY inhibition is also corroborated by the increase in apoptosis of MYCN-amplified neuroblast cells [57]. Of note, the first evidence on the role of this enzyme as a key molecule in the progression of MYCN-amplified neuroblastoma dates back to the early 1990s when several studies in different NB mouse models were employed [58–62] More recently, considering the potential implication of this gene on the clinical management of NB, Novak and colleagues [63] hypothesized that the identification of its genetic variations may have significant impact during development of the recurrent or progressive disease. Non-synonymous variants in AHCY gene have been found for example to contribute to the slow progression of the disease, even in more aggressive cases. It affects the maintenance of the catalytic capacity of AHCY, leading to the consequent functional effects in NB patients. Thus, also the potential use of AHCY variants may constitute a molecular biomarker. Finally, it is known that MYCN-amplification alters key metabolic pathways as glycolysis and gluconeogenesis. Oliynyk et al. [64] found that the stressed phenotype of MYCN amplified NB cells was characterized by a shift in the metabolic balance toward robustly increased oxidative phosphorylation as well as enhanced aerobic glycolysis. Interestingly, AHCY has been recently included in the *glycosyl compound metabolic process* gene set, suggesting that AHCY might link glucose with adenosine or homocysteine [65] and alter this metabolic process. This data suggest that, beyond their correlation, these genes are effectively functionally interconnected to each other.

**DPYSL3 and neuroblastoma**

DPYSL3 (also referred to as collapsing response mediator protein 4, CRMP4) is a member of the DPYSL gene family, highly expressed in developing and adult nervous systems. It functions in a variety of cellular processes, including cell migration, differentiation, neurite extension, and axonal regeneration. It has been reported to be involved also in the metastatic process of tumor cells [66]. Some authors debrided DPYSL3 as a metastasis suppressor, while others [67] reported it facilitates pancreatic cancer cell dissemination via a strong interaction with other cell adhesion factors, including Ezrin, focal adhesion kinase and c-SRC. In breast cancer, DPYSL3 knockdown determined a reduced proliferation, but a still enhanced motility and increased expression of epithelial-to-mesenchymal transition markers, suggesting that DPYSL3 is a multifunctional signaling modulator. In neuroblastoma cells, it has been shown that DPYSL3 regulates the actin cytoskeleton, whose dynamic reorganization is known to be fundamental for cell migration. DPYSL3 was found abundant in the cytosol of B35 neuroblastoma cells and to co-localizes with F-actin in regular rib-like structures within lamellipodia of these cells, with which physically interacts. The critical functional equilibrium between DPYSL3 and F-actin is demonstrated by the fact that DPYSL3 overexpression inhibited the migration of B35 neuroblastoma cells, while its knockdown enhanced cell migration and disturbed rib-like actin-structures in lamellipodia [68]. Interestingly, studies using genetic approaches showed that DPYSL3 levels were inversely altered with changes in MYCN expression, thus suggesting a MYCN negative regulation of DPYSL3 in NB cells, probably via EZH2. This negative regulation may be also mediated by GSK3b [69]. The regulation of DPYSLs by GSK-3b in neuronal polarity or axon outgrowth has already been reported. Moreover, it is known that Akt in NB cells phosphorylate and thus inactivate GSK-3b [70] and that inactivation of GSK-3b would lead to an increase of MYCN protein expression. Thus increased MYCN levels, via amplification or as the result of Akt-mediated GSK-3b inactivation would lead to DPYSL3 suppression in NB cells. This mechanistic evidence also support an important prognostic role for DPYSL3 expression. Indeed, tumors of advanced-stage NB patients have a good prognosis if characterized by elevated levels of DPYSL3, and even in high-risk NB patients the levels of DPYSL3 mark those patients who may have a better overall survival [71].

**NME1 and neuroblastoma**

The NME1 gene is located in the 17q21.3 region, whose gain is a common evaluated factor predicting adverse clinical outcome in neuroblastoma patients. NME1 has been shown to be involved in multiple critical cellular behaviors, including cell proliferation, differentiation, and neural development. In cancer, its overexpression has a dichotomous role as both a suppressor and a promoter of tumor metastasis, based on the cancer type. In breast and prostate carcinomas for example, high levels of this protein is associated with good survival and low-risk features. In contrast, in pediatric cancer such as neuroblastoma, it correlates with aggressive neuroblastoma tumor features while increased NME1 expression has been identified as a component of gene expression, signatures most significantly associated with poor neuroblastoma patient outcomes. Some authors attribute this evidence to the histidine kinase capacity of NME1 that catalyze transfer of the activated phosphate from the autophosphorylated histidine 118 residue (H118) onto target proteins. It is plausible to assume that this results in an increased activity of proteins involved in cell migration and differentiation. Indeed, NME1 shRNA knock-down disrupts differentiation of neuroblastoma cells induced by 13-cis-retinoic acid (CRA) treatment [72]. Carotenuto et al. [73] demonstrated that NME1 form a protein complex with h-Prune, trough which could act as a pro-metastatic gene. As a matter of fact, the overexpression of NME1 and h-Prune enhances the aggressiveness of NBL cells both in vitro and in vivo. Thus, the disruption of this interaction might constitute a potential therapeutic intervention for neuroblastoma patients [73]. Moreover, A Negroni et al. [74], discovered that patients bearing S120G mutation in NME1 gene have worst prognosis respect to wild type ones. It has been demonstrated that NME1-S120G is more effective in promoting cell invasiveness and metastasis of neuroblastoma in vitro and in vivo. S120G may be defined as a gain-of-function mutation, since it increases the invasiveness not only of neuroblastoma, but also of breast and prostate carcinoma cells. An apparent gain-of-function of the S120G mutation of NME1 is likely caused by a protein-folding defect, which affects its protein-protein interactions. However, the molecular mechanism(s) by which NME1 promotes neuroblastoma metastasis still remains elusive [74]. Finally, Okabe-Kado et al [75] examined serum NME1 protein levels in 217 patients with neuroblastoma, demonstrating that (i) the serum NME1 protein level was higher in neuroblastoma patients than in control children; (ii) patients with MYCN amplification had higher serum NME1 levels than those with a single copy of MYCN. Thus, the detection of serum NME1 protein levels, contributing to predictions of clinical outcome in patients with neuroblastoma, may be also proposed as not invasive prognostic markers [75].

## Discussion

**The prognostic role of AHCY, DPYSL3, and NME1** The evaluation of the impact of AHCY, DPYSL3 and NME1 on the prognosis of patients affected by Neuroblastoma showed the importance of these genes in terms of association with the death rate (Fig. 1) and prediction of the survival probabilities across time (Table 4). Despite the limited number of studies able to provide a high number of patients with available survival data, both observed risk and hazard ratios suggested that low levels of DPYSL3 expression and high levels of AHCY and NME1 expressions shorten overall survival, even taking in consideration potential confounders like the age of the patients. The combination of these genes showed that, except for one dataset, in all the other studies the combined effect of these genes showed significant hazard ratios (*p*-values $\le 0.001$). Only for TARGET data the *p*-value was not significant, but $<0.01$. The evaluation of the pooled risk ratios showed high risk ratios for the combined score ($>8.5$). Despite the heterogeneity was high ($I^{2}$=74%), no significant biases across the studies were observed from the funnel plots (Begg and Mazumdar’s test *p*-value $>0.05$, Fig. S1). Similar results were observed also when the three genes were analyzed independently. Considering the potential overlap between the datasets used in our analysis (Table 3) and those used in the studies of the prognostic signatures reported in Table 1, we observed that the number of samples in each study does not exceed the 40% of overlap with respect to the total number of samples considered in our analysis, i.e. 1,648, as reported in the Supplementary Table S1. In addition, except for AHCY which was detect in [8], showing an overlap equal to 32.3% with our samples, the other studies where we found the three genes show an overlap below 15.5%.

Moreover, we verified the predictive power of the three genes employed with machine learning to an independent gene expression dataset. Our three proposed methods and Random Forests were able to correctly classify survived patients with neuroblastoma and deceased patients with the same disease with a high accuracy, reaching MCC = +0.517 and ROC AUC = 0.877 on average. This binary classification result confirms the prognostic ability of these three genes.

We then performed a thorough literature search where we retrieved tens of peer-reviewed published studies associating each of our proposed prognostic genes with neuroblastoma survival. We did not only look for the ACHY, DPYSL3, and NME1 gene names, but also their aliases found on GeneCards.org. Our brief review of this biomedical literature confirmed a strong association between the three prognostic genes and neuroblastoma development. This confirmation comes with no surprise, since we selected our three prognostic genes from ten signatures already available in the biomedical literature.

These results confirm the robustness of the three proposed prognostic genes, that we validated in this study in three different ways (statistical analysis, machine learning analysis, and literature review). Since the prognostic signatures for neuroblastoma have minimal overlap, indicating that most genes are prognostically relevant mainly in one single study, our outcomes result being particularly relevant and interesting in oncologic research.

It is also relevant to notice that the NME1 is present as prognostic gene in three signatures’ studies [10, 11, 14] which employ the GSE3960 and E-TABM-38 datasets, among others.

**Conclusions** To the best of our knowledge, there are no studies on neuroblastoma reviewing the current status of the associated genetic signatures available in the literature. Here, we showed the low overlap among the prognostic genetic signatures provided so far and highlighted the most recurrent genes detected by the neuroblastoma studies, We then validated these genes using the largest gene expression datasets from the public repositories in order to provide an evaluation of the prognostic impact at high accuracy on the highest number of samples available. Our results pointed out the relevant impact of the genes AHCY, DPYSL3 and NME1 on neuroblastoma prognosis as future targets for future neuroblastoma genetics studies and the development of novel therapies.

## Supplementary Information

**Additional file 1.** Supplementary information.

## Data Availability

The gene expression datasets employed in this study can be freely and openly found at the following URLs: $\bullet$ https://www.ncbi.nlm.nih.gov/geo/query/acc.cgi?acc=GSE16237 $\bullet$ https://www.nature.com/articles/s41467-021-21247-8#data-availability $\bullet$ https://www.ncbi.nlm.nih.gov/geo/query/acc.cgi?acc=GSE16476 $\bullet$ https://www.ncbi.nlm.nih.gov/geo/query/acc.cgi?acc=GSE62564 $\bullet$ https://www.ebi.ac.uk/arrayexpress/experiments/E-MTAB-8248 $\bullet$ https://www.ebi.ac.uk/arrayexpress/experiments/E-MTAB-38 $\bullet$ https://www.ncbi.nlm.nih.gov/geo/query/acc.cgi?acc=GSE85047 $\bullet$ https://www.ebi.ac.uk/arrayexpress/experiments/E-MTAB-179 $\bullet$ https://www.ncbi.nlm.nih.gov/geo/query/acc.cgi?acc=GSE16716

## Abbreviations

AHCY: Adenosylhomocysteinase
CI: Confidence Interval
CRA: Cis-retinoic acid
DPYSL3: Dihydropyrimidinase Like 3
GEO: Gene Expression Omnibus
HR: Hazard Ratio
LR: Log-Rank
MCC: Matthews correlation coefficient
NME1: NME/NM23 Nucleoside Diphosphate Kinase 1
NB: Neuroblastoma
*p*-value: Probability value
RR: Risk ratio
