# Supplementary material for: Signature literature review reveals AHCY, DPYSL3, and NME1 as the most recurrent prognostic genes for neuroblastoma
Source: BioData Min. 2023 Mar 4;16:7. doi: 10.1186/s13040-023-00325-1 (PMC9985261; doi:10.1186/s13040-023-00325-1)
Supplement: Supplementary file 1 — Additional file 1. Supplementary information. [file 13040_2023_325_MOESM1_ESM.pdf]

S1 Supplementary information

S1.1 Neuroblastoma prognostic signature search

We performed this search on Google Scholar [43] on 22nd February 2022 by using the following keywords:

`neuroblastoma prognostic signature`
`neuroblastoma prognosis signature`
`neuroblastoma genetic signature -diagnosis -diagnostic`

S1.2 Genes of the prognostic signatures

Cangelosi et al. [7]: AK4, EGLN1, FAM162A, MTFP1, PDK1, PGK1.

Zhong et al. [8]: CDK4, PIK3R1, THRA, MAP2K2, ULBP2.

Jin et al. [9]: AMH, IGHV3-20, IGKV1-16, IRF7, KLRK1, MARCO, SECTM1, SOCS1, UNC93B1.

Vermeulen et al. [10]: AHCY, AKR1C1, ARHGEF7, BIRC5, CADM1, CAMTA1, CAMTA2, CD44, CDCA5, CDKN3, CHD5, CLSTN1, CPSG3, DDC, DPYSL3, ECEL1, ELAVL4, EPB41L3, EPHA5, EPN2, FYN, GNB1, HIVEP2, INPP1, MAP2K4, MAP7, MAPT, MCM2, MRPL3, MTSS1, MYCN, NHLH2, NME1, NRCAM, NTRK1, ODC1, PAICS, PDE4DIP, PIK3R1, PLAGL1, PLAT, PMP22, PRAME, PRDM2, PRKACB, PRKCZ, PTN, PTPRF, PTPRH, PTPRN2, QPCT, SCG2, SLC25A5, SLC6A8, SNAPC1, TNFRSF25, TYMS, ULK2, WSB1

De Preter et al. [11]: AHCY, AKR1C1, ARHGEF7, BIRC5, CADM1, CAMTA2, CDCA5, CDKN3, CLSTN1, DDC, DPYSL3, ECEL1, EPB41L3, EPHA5, EPN2, FYN, GNB1, HIVEP2, INPP1, MAP7, MAPT, MCM2, MRPL3, MYCN, NCAN, NME1, NRCAM, NTRK1, ODC1, PAICS, PLAGL1, PMP22, PRKACB, PRKCZ, PTN, PTPRN2, SCG2, SLC25A5, SNAPC1, TYMS, ULK2, WSB1.

Valentijn et al. [12]: ACSL4, AGPAT4, ANAPC1, ANO4, ARHGEF3, ARL4D, ASTN1, ATAD2, C10orf26, C19orf43, C1orf114, C1orf97, C20orf72, C4orf34, C5orf41, C7orf60, C9orf95, CAMK2N1, CCNI, CELF3, CLU, CNNM1, CNTN1, CRTAP, CSRNP3, CYP4V2, DCTPP1, DHCR7, DIMT1L, DIRAS3, DKC1, DNER, E2F5, E2F8, ECE2, EIF2C4, EIF2S2, EPB41L1, FAM134B, FAM167A, FAM174A, FAM190B, FAM36A, FAM82A2, FAM85A, FOXRED2, GABRA5, GAR1, GNAZ, GPR85, GPX7, GRSF1, GTPBP1, HJURP, HMP19, HPDL, IARS, IFT20, IMPDH2, IQCG, KIAA0226, KIAA0895, KIF23, KIF4A, KLHL14, LIG3, LOC442075, LRRC59, LYAR, LYSMD2, MAP2, MBNL2, MCCC2, MCM3, MCM5, METTL9, MFAP4, MFSD6, MKRN1, MRPL45, MSH6, MTAP, MYPOP, NAT15, NCAPH, NOC4L, NRBP2, NT5DC3, NUDT14, OIP5, OSCP1, PCCB, PDE5A, PFKM, PHF20, PHGDH, PKIA, PLEKHA3, PMM2, POLA2, POLD2, POLE2, PRDX6, PREP, PRMT1, PRMT7, PUS7, PYCR1, RAB6B, RAD51, RAD51AP1, RAD54L, RAGE, RAVER1, RAVER2, RHOBTB3, RHOC, RND2, RNF144A, RNF146, RRM2, RUNDC3A, SCN3A, SESTD1, SH3BGR, SH3BGRL2, SKA3, SLBP, SLC20A1,

SRF, STMN2, SUSD5, SYNPO2, SYT13, SYT4, TBX2, TCTN3, TEX15, TIPIN, TMEM150C, TMEM170B, TP53INP1, TPPP3, TRAK2, TRIM69, UBE2H, UBL7, UBQLN2, WDR18, WDR76, WDR77, XPO5, ZBTB4, ZNF25, ZNF317, ZNF608, ZNRF3.

Zhong et al. [13]: AHCY, ERCC6L, NCAN, STK33.

Garcia et al. [14]: CHD5, NME1, PAFAH1B1.

Frumm et al. [15]: ABCB1, ADD3, ARHGEF3, ASCL1, CACNA2D2, CALML4, CDKN1A, CHGA, CHST5, CPSF1, CRABP2, CRH, CTSB, CYP26A1, DDAH2, DDEF2, DLK1, DOCK4, DPYSL3, DUSP6, EGR1, FLJ10357, FLJ12584, FLNB, GATA3, HEBP2, HERC2, HOXD4, HS2ST1, IER3, IGFBP5, ISGF3G, JARID2, KCTD12, KCTD13, MAFF, MMP11, MTMR1, N4BP1, OIP106, PELO, PLAT, PLEKHA6, PLK2, PPIF, PRKCH, PTBP1, RAB20, RAP80, RBP1, RGS16, SLC29A1, ST6GAL1, STX6, SYNJ2, SYT11, TGM2, TPST1, VGF.

Wang et al. [16]: HNRNPC, IGF2BP2, METT14, WTAP, YTHDF1.

S1.3 Additional details on validation results

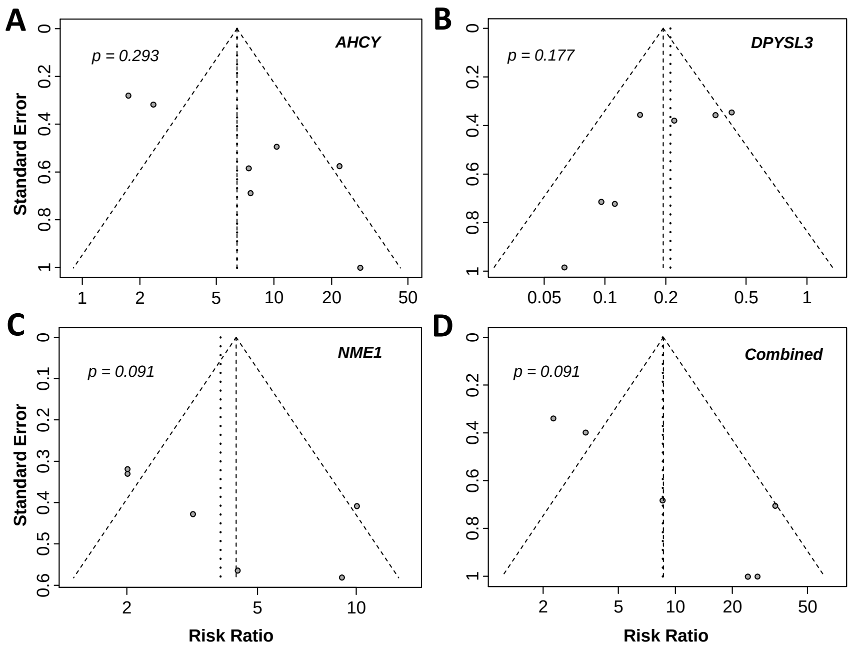

Figure S1: ***Funnel plots indicating that the publication bias was unlikely for the relative risk in all-cause mortality****. Funnel plots for the three genes considered separately, i.e. AHCY (A), DPYSL3 (B), NME1 (C), and for the score which combines the expression-based ranks of the three genes, considering the reversed rank for DPYSL3 since it showed RRs < 1. p-values resulting from Begg and Mazumdar's tests are displayed at the top-left side of each panel. Abbreviations: RR, Risk Ratio; CI, Confidence Interval.*

study	datasets used	samples in common with our analysis	overlap percentage
Vermeulen et al. [10]	GSE2283, GSE3960, E-TABM-38, E-MEXP-669 E-MEXP-83, Berwanger et al. 2002	251	15.2%
De Preter et al. [11]	GSE2283, GSE3960, E-TABM-38, Berwanger et al. 2002	251	15.2%
Garcia et al. [14]	GSE3960, E-TABM-38	251	15.2%
Valentijn et al. [12]	GSE16476, GSE13136, GSE16237, GSE12460, E-TABM-38	339	20.6%
Frumm et al. [15]	GSE45587	0	0.00%
Zhong et al. [13]	GSE49710, GSE45480, GSE16476	88	5.40%
Wang et al. [16]	GSE49711, TARGET	161	9.80%
Cangelosi et al. [7]	GSE62564, GSE120572, GSE16476, GSE13136, E-MTAB-1781, GSE16237	586	35.6%
Jin et al. [9]	GSE62564, TARGET	659	40.00%
Zhong et al. [8]	GSE49710, GSE16476, TARGET, GSE85047	532	32.3%

Table S1: ***Overlap between the neuroblastoma studies of the prognostic signatures found in the literature and the datasets considered in our analysis..*** *The percentage is calculated dividing the number of samples in common and the total number of samples considered in our analysis, that are 1,648 samples.*
